# Comparing the effects of transthoracic echocardiography and transesophageal echocardiography on stress injury, pain mediators in patients with severe aortic stenosis

**DOI:** 10.5937/jomb0-55774

**Published:** 2025-06-13

**Authors:** Aidong Chen, Bin Chen, Po Yang, Xiaoming Shi, Zhipeng Xu, Fanxin Deng

**Affiliations:** 1 The First Affiliated Hospital of Nanjing Medical University, Department of Cardiovascular Surgery, Nanjing, Jiangsu, China; 2 Nanjing Medical University, Sir Run Run Hospital, Department of Cardiothoracic Surgery, Nanjing, Jiangsu, China

**Keywords:** transesophageal echocardiography, aortic stenosis, transthoracic echocardiography, inflammatory factors, stress response, hemodynamics, pain mediators, transezofagealna ehokardiografija, aortna stenoza, transtorakalna ehokardiografija, upalni faktori, odgovor na stres, hemodinamika, medijatori bola

## Abstract

**Background:**

We compared the differences in the effects of transthoracic echocardiography (TTE) and transesophageal echocardiography (TEE) on hemodynamics, inflammatory stress response, and pain mediators in patients with severe aortic stenosis (AS).

**Methods:**

204 patients with severe AS treated with transcatheter aortic valve replacement (TAVR) in our hospital were selected as the research subjects from January 2022 to February 2024. Among them, 109 patients received TTE (TTE group), and another 95 received TEE (TEE group). Differences in the evaluation effects of preoperative echocardiography and multi-slice helical computed tomography (MSCT) in all patients were compared, and changes in echocardiographic parameters before and after surgery were observed. In addition, the differences in postoperative hemodynamics, cardiac function [brain natriuretic peptide (BNP), cardiac troponin I (cTnI), creatine kinase isoenzyme (CK-MB)], stress response [superoxide dismutase (SOD), malondialdehyde (MDA)], inflammatory factors [Interleukin-1b/6 (IL-1b/6), tumour necrosis factor-a (TNF-a)], and pain mediators [5-hydroxytryptamine (5-HT), endothelin-1 (ET-1), prostaglandin E2 (PGE2), substance P (SP)] between the observation and TTE groups were compared.

**Results:**

No differences were identified in the evaluation of the aortic root between echocardiography and MSCT (P>0.05). After surgery, parameters such as LVESD and IVST decreased, while LVEF and AVA increased (P<0.05). The TEE group showed superior postoperative hemodynamics to the TTE group (P<0.05). There was no difference in cardiac function between the two groups (P>0.05), but IL-1b, IL-6, TNF-a, 5-HT, ET-1 and SP were lower in the TEE group than in the TTE group, whereas SOD was higher than in the TTE group (P<0.05).

**Conclusions:**

TTE and TEE have an excellent guiding effect on the implementation of TAVR in patients with severe AS, among which TEE is more helpful in improving the effectiveness and safety of TAVR.

## Abstract (sr)

**Uvod:**

Upoređeni su efekti transtorakalne ehokardiografije (TTE) i transezofagealne ehokardiografije (TEE) na hemodinamiku, upalni odgovor na stres i medijatore bola kod pacijenata sa teškom stenozom aorte (AS).

**Metode:**

U istraživanje je uključeno 204 pacijenata sa teškom AS, lečenih transkateterskom ugradnjom aortnog zaliska (TAVR) u našoj bolnici od januara 2022. do februara 2024. godine. Među njima, 109 pacijenata je bilo podvrgnuto TTE (TTE grupa), a 95 pacijenata TEE (TEE grupa). Upoređeni su efekti preoperativne evaluacije pomoću ehokardiografije i višeslojne spiralne kompjuterizovane tomografije (MSCT) kod svih pacijenata, kao i promene ehokardiografskih parametara pre i posle operacije. Pored toga, analizirane su razlike u postoperativnoj hemodinamici, funkciji srca [Moždani natriuretski peptid (BNP), srčani troponin I (cTnI), kreatin kinaza MB (CK-MB)], odgovoru na stres [superoksid dismutaza (SOD), malondialdehid (MDA)], upalnim faktorima [interleukin-1b/6 (IL-1b/6), faktor nekroze alfa (TNF-a)] i medijatorima bola [5-hidroksitriptamin (5-HT), endotelinski faktor-1 (ET-1), prostaglandin E2 (PGE2), supstanca P (SP)] između TTE i TEE grupa.

**Rezultati:**

Nisu utvrđene razlike u proceni aortnog korena između ehokardiografije i MSCT (P>0,05). Nakon operacije, parametri poput LVESD i IVST su se smanjili, dok su LVEF i AVA porasli (P<0,05). TEE grupa je pokazala bolju postoperativnu hemodinamiku u odnosu na TTE grupu (P<0,05). Nije bilo razlika u funkciji srca između dve grupe (P>0,05), ali su vrednosti IL-1b, IL-6, TNF-a, 5-HT, ET-1 i SP bile niže u TEE grupi nego u TTE grupi, dok je nivo SOD bio viši u TEE grupi (P<0,05).

**Zaključak:**

TTE i TEE imaju odličan usmeravajući efekat na sprovođenje TAVR kod pacijenata sa teškom AS, pri čemu je TEE korisnija za poboljšanje efikasnosti i bezbednosti TAVR.

## Introduction

Aortic stenosis (AS) is the most frequent valvular heart disease among older individuals [1]. AS can present as dyspnoea, angina, syncope and palpitations [2]. Statistics show that about 12 per cent of people aged 75 have AS [3].

Surgical aortic valve replacement (SAVR) is the primary clinical treatment for severe AS, which replaces the original diseased or abnormal heart valves with artificial valves to restore normal cardiac function [4]. However, there are still some severe AS patients who cannot tolerate SAVR with significant trauma due to various reasons such as advanced age, left ventricular dysfunction, pulmonary insufficiency, or comorbidities [5]. With the development of medical technology, transcatheter aortic valve replacement (TAVR) has gradually become the preferred treatment for severe AS due to its advantages of short operation time, less trauma, and quick recovery [6] [7]. In TAVR, echocardiography is one of the essential means for preoperative screening, intraoperative monitoring, and postoperative follow-up, which can quickly evaluate the significance of essential indexes such as left ventricular function [8]. Currently, the commonly used clinical ultrasound protocols in intermediate TAVR are transthoracic echocardiography (TTE) and transesophageal echocardiography (TEE), both of which can accurately reflect the patient’s cardiac status and help the clinic perform TAVR better. However, we found that all the related studies focused on TAVR, often ignoring the importance of imaging technology, with little research discussing the application differences of TTE and TEE in TAVR. Therefore, the use of both protocols remains highly controversial. For example, Rozenbaum Z et al. [9] concluded that TTE provides a more accurate indication of vascular resistance in the patient’s lungs and is more conducive to controlling blood loss during surgery. In contrast, Bax JJ et al. [10] stated that TEE is more accurate in assessing cardiac function in complex clinical situations.

To address these limitations, this study will analyse the use of echocardiography in TAVR to identify which ultrasound protocol is more suitable. Thus, it will further improve the surgical safety and prognosis of TAVR for severe AS.

## Materials and methods

### Research participants

Two hundred and four patients with severe AS admitted to our hospital from January 2022 to February 2024 were selected as the research participants. Age 58–85 years, mean (72.26±5.11) years; disease duration 2–8 years, mean (4.61±1.18) years; 124 males, 80 females; New York Heart Association (NYHA) [11] grade III 165 cases, grade IV 39 cases. All of them completed TAVR in our hospital, of which 109 underwent transthoracic echocardiography (TTE) as the TTE group, and 95 underwent transesophageal echocardiography (TEE) as the TEE group. This study was approved by the Ethics Committee of our hospital (No. 2021-SR-020) and strictly followed the *Declaration of Helsinki*. All study subjects signed an informed consent form.

### Inclusion and exclusion criteria

Inclusion criteria: (1) age>18 years old, with complete medical records; (2) severe AS [maximum aortic valve orifice blood flow velocity (V_max_) 4.0 m/s, mean aortic valve pressure gradient (AVPG_mean_)40 mmHg, or aortic valve area (AVA)<1.0 cm^2^; NYHA cardiac functional classification >grade II] diagnosed by our hospital. (3) meeting the indications for TAVR surgery and completing surgical treatment in our hospital. Exclusion criteria: (1) inability to undergo echocardiography (including elderly patients who are unable to hold their breath to acquire three-dimensional images); (2) allergies or contraindications to anticoagulant/antiplatelet therapy; (3) cerebrovascular accident or transient ischemic attack within 2 months before surgery; (4) severe liver, lung, and kidney diseases and contrast agent allergies; (5) estimated survival after correction of AS<12 months.

### Surgical procedure

Before surgery, the patient fasted, abstained from food and drink, and completed all routine tests. The TAVR for all patients was performed by the same physician in our hospital. By combining preoperative multi-slice helical CT (MSCT) (Somatom Sensation 16, Siemens, Germany) and echocardiography to measure the valve annulus diameter, the patient was fitted with a suitable type of prosthetic valve. A stiffened guidewire was fed into a valve-equipped catheter delivery system to the aortic annulus, where the valve was released with the assistance of aortic root angiography and rapid right ventricular pacing (frequency 120–150 beats/minute, pacing time: 10-20 seconds). Immediately after the operation, echocardiography was performed to evaluate prosthetic aortic valve positioning and perivalvular leakage (PVL). After surgery, patients must avoid a high-fat, high-salt and high-sugar diet and consume plenty of vegetables and fruits. Medications such as anticoagulants and antiplatelet agents are taken as prescribed to prevent thrombosis and reduce the risk of cardiovascular events. Clinical success was defined as no intraoperative transfer to surgical thoracotomy, no prosthetic valve displacement or detachment, moderate or higher PVL or coronary artery occlusion within 30 days postoperatively, and no implantation of a permanent pacemaker [11].

### Imaging examination methods

All patients were examined by MSCT 3–5 days before surgery. Experts in the TAVR team analysed the maximum diameter, minimum diameter, area, and circumference of the aortic valve annulus and the height of the openings of the left and right coronary arteries to select an appropriate valve model. In addition, echocardiography was performed before the operation and 30 days after surgery. TTE group: TTE was employed, with a probe frequency of 2.0–4.0 MHz. TEE group: TEE was utilised, with a probe frequency of 5 MHz. The examination instrument was a Philips EPIIQ 7 colour Doppler ultrasound (Nether lands). Under the display of four-chamber cardiac images, three short-axis images of the apical two-chamber and the basal, middle, and apical segments of the left ventricle in three cardiac cycles were collected and saved.

### Endpoints

Surgical outcomes were analysed. (2) Differences in the detection results of the maximum diameter, minimum diameter, circumference, area, and aortic valve area (AVA) of the aortic root between MSCT and echocardiography before surgery in all patients were observed. (3) Changes in echocardiographic parameters before and after surgery in patients, including left atrial diameter (LAD), left ventricular end-diastolic diameter (LVEDD), left ventricular end-systolic diameter (LVESD), left ventricular ejection fraction (LVEF), interventricular septum thickness (IVST), posterior wall thickness (PWT), pulmonary artery systolic pressure (PASP), maximum aortic valve pressure gradient (AVPG_max_), AVPG_mean_, V_max_, and AVA, were determined. (4) Differences in postoperative hemodynamics between the observation and TTE groups, including systemic vascular resistance index (SVRI), global end-diastolic volume index (GEDV), intrathoracic blood volume index (ITBVI), and extravascular lung water index (EVLWI), were analysed. (5) Venous blood was collected from patients after examination for laboratory tests, specifically cardiac function [Brain natriuretic peptide (BNP), cardiac troponin I (cTnI), Creatine kinase iso enzyme (CK-MB)], stress response [Superoxide dismutase (SOD), Malondialdehyde (MDA)], inflammatory factors [Interleukin-1β/6 (IL-1β/6), Tumor necrosis factor-α (TNF-α)], and pain mediators [5-hydrox ytryptamine (5-HT), Endothelin-1 (ET-1), Prostaglandin E_2_ (PGE_2_), Substance P (SP)]. These tests were done by Sir Run Run Hospital, Nanjing Medical University medical laboratory.

### Statistical analysis

SPSS26.0 statistically analysed all the data in this study. Count data were recorded as [n(%)], and the chi-square test was used to compare groups. Measurement data, described as (x̄±s), were compared between groups with the independent sample t-test. *P*<0.05 was the statistical significance level.

## Results

### Surgical conditions of patients

**Figure 1:**
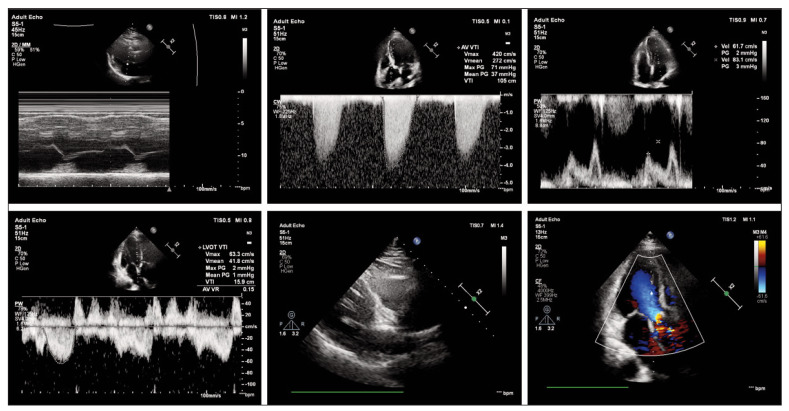
Echocardiographic findings of a patient. Female, 71 years old.

All patients were treated via the transfemoral approach, with 16 cases implanted with VenusA valves and 31 with VitaFlow valves. The operation was successful in 39 cases. Seven patients developed grade III atrioventricular block within one week after surgery and underwent permanent pacemaker implantation; one patient underwent coronary artery bypass grafting due to mechanical coronary occlusion caused by valve displacement. None of the patients experience moderate or above PVL. Figure 1 demonstrates the patient’s echocardiographic findings.

### Evaluation effect of echocardiography and MSCT on the aortic root

Regarding the aortic valve annulus, we found no significant differences between echocardiography and MSCT in measuring the maximum diameter, minimum diameter, circumference, and area (*P*>0.05). In addition, the measurement result of AVA by echocardiography was (0.67±0.15) cm^2^, and that by MSCT was also (0.69±0.16) cm^2^, also without statistical significance (*P*>0.05) (Table 1).

**Table 1:** Evaluation effect of echocardiography and MSCT on the aortic root.

n=204	Maximum diameter<br>(mm)	Minimum Diameter<br>(mm)	Area (mm^2^)	Circumference<br>(mm)	AVA<br>(cm^2^)
MSCT	27.32±3.33	22.19±3.56	485.83±169.93	78.31±11.68	0.69±0.16
Echocardiography	27.78±4.07	22.55±3.08	474.06±141.20	79.12±10.66	0.67±0.15
t	1.252	1.116	0.760	0.731	0.901
*P *	0.211	0.265	0.448	0.465	0.368

### Changes in echocardiographic parameters before and after surgery

Statistics of echocardiographic parameters showed that LAD and LVEDD did not change significantly before and after surgery (*P*>0.05); however, LVESD, IVST, PWT, PASP, AVPG_max_, AVPG_mean_, and V_max_ were all decreased after the operation compared to the levels before surgery, while LVEF and AVA were increased (*P*<0.05) (Table 2).

**Table 2:** Changes in echocardiographic parameters before and after surgery.

n=204	Before surgery	After surgery	t	* P *
AVPG_max_ (m/s)	4.82±0.76	2.34±0.76	33.120	<0.001
AVPG_mean_ (mmHg)	53.99±15.79	11.96±7.11	34.660	<0.001
V_max_ (m/s)	4.99±0.75	2.28±0.47	43.840	<0.001
LAD (mm)	43.95±6.59	42.81±8.12	1.691	0.092
LVEDD (mm)	49.67±7.29	48.50±6.76	0.373	0.710
LVESD (mm)	35.08±8.91	29.39±6.92	7.213	<0.001
IVST (mm)	13.74±2.00	12.25±1.30	8.905	<0.001
PWT (mm)	12.86±1.34	11.35±1.31	11.480	<0.001
PASP (mmHg)	49.14±5.74	38.95±8.44	8.156	<0.001
LVEF (%)	57.37±12.44	37.83±6.02	20.190	<0.001
AVA (cm^2^)	0.67±0.15	1.81±0.24	58.910	<0.001

### Baseline data of patients

We compared patients’ clinical baseline data and found no statistically significant differences in age, sex, and course of disease between the TEE group and the TTE group (*P*>0.05), confirming the comparability (Table 3).

**Table 3:** Baseline data of patients.

	TTE group (n=109)	TEE group (n=95)	t (or χ^2^)	* P *
Age	72.54±5.66	72.16±4.42	0.534	0.594
Sex			0.130	0.718
male	65 (59.63)	59 (62.11)		
female	44 (40.37)	36 (37.89)		
Course of disease (years)	4.72±1.23	4.48±1.11	1.457	0.147
NYHA cardiac function grade			0.431	0.512
IV	19 (17.43)	20 (21.05)		
III	90 (82.57)	75 (78.95)		
Combined hypertension			0.113	0.737
yes	71 (65.14)	64 (67.37)		
no	38 (34.86)	31 (32.63)		
Combined diabetes mellitus			0.543	0.370
yes	62 (56.88)	50 (52.63)		
no	47 (43.12)	45 (47.37)		
Smoking			1.317	0.251
yes	33 (30.28)	36 (37.89)		
no	76 (69.72)	59 (62.11)		
Drinking			0.529	0.396
yes	27 (24.77)	20 (21.05)		
no	82 (75.23)	75 (78.95)		

### Differences in hemodynamics between TEE and TTE

Comparing the hemodynamics between the TEE group and the TTE group, it can be seen that the SVRI, GEDVI, and ITBVI in the TEE group were (2200.38±586.30) dyn·s·cm^5^/m^2^, (838.43±179.51) mL/m^2^, and (1052.81±191.67) mL/m^2^, respectively, which were all higher compared to the TTE group (*P*<0.05); the EVLWI of the TEE group was (11.65±4.86) mL/kg, which was even lower compared with the TTE group (*P*<0.05) (Table 4).

**Table 4:** Differences in hemodynamics between TEE and TTE.

Groups	SVRI (dyn·s·cm5/m^2^)	GEDVI (mL/m^2^)	EVLWI (mL/kg)	ITBVI (mL/m^2^)
TTE (n=109)	1657.22±541.66	633.50±174.78	15.92±6.13	963.14±207.77
TEE (n=95)	2200.38±586.30	838.43±179.51	11.65±4.86	1052.81±191.67
t	6.875	8.249	5.450	3.187
* P *	<0.001	<0.001	<0.001	0.002

### Differences in cardiac function between TEE and TTE

However, in the comparison of cardiac function, we found no statistically significant differences in BNP, cTnI, and CK-MB between the TTE and TEE groups (*P*>0.05), suggesting that the effects of TTE and TEE on cardiac function were similar (Table 5).

**Table 5:** Differences in cardiac function between TEE and TTE.

Groups	BNP (pg/mL)	cTnI (ng/mL)	CK-MB (U/L)
TTE (n=109)	151.67±37.28	1.01±0.24	52.54±13.98
TEE (n=95)	156.77±39.85	1.08±0.36	50.79±12.05
t	0.944	1.617	0.951
* P *	0.346	0.107	0.343

### Differences in stress injuries and inflammatory factors between TEE and TTE

In contrast, in comparing stress injury and in flammatory factors, we found no difference in the com parison of MDA between the two groups (*P*>0.05). Still, the SOD in the TEE group was higher than that in the TTE group, while IL-1β, IL-6, and TNF-α were lower than that in the control group (*P*<0.05), which showed that stress injury and inflammatory response were milder in the TEE group (Table 6).

**Table 6:** Differences in stress injuries and inflammatory factors between TEE and TTE.

Groups	SOD (U/L)	MDA (mmol/L)	IL-1β (pg/mL)	IL-6 (pg/mL)	TNF-α (pg/mL)
TTE (n=109)	6.98±2.65	171.32±28.71	25.63±4.95	18.99±4.26	23.18±4.61
TEE (n=95)	7.83±3.14	173.72±24.76	21.51±5.52	15.28±3.05	19.17±2.02
t	2.075	0.636	5.618	7.055	7.841
* P *	0.039	0.525	<0.001	<0.001	<0.001

### Differences in pain between TEE and TTE

Finally, we assessed the pain in both groups by examining the pain mediators, and it was seen that there was no difference in the comparison of PGE_2_ between the two groups as well (*P*>0.05). However, 5-HT, ET-1 and SP were lower in the TEE group than in the TTE group (*P*<0.05), suggesting that the pain in patients in the TEE group was lower than that in the TTE group (Table 7).

**Table 7:** Differences in pain between TEE and TTE.

Groups	5-HT (ng/L)	ET-1 (ng/L)	PGE2 (ng/L)	SP (ng/L)
TTE (n=109)	54.71±8.84	0.55±0.10	80.85±0.87	5.01±1.02
TEE (n=95)	51.77±7.93	0.52±0.11	79.67±9.79	4.51±1.24
t	2.485	2.450	0.912	3.159
* P *	0.014	0.015	0.363	0.002

## Discussion

In this study, we found that compared to TTE, TEE reduces stress response and pain mediators in patients with severe AS with a higher safety profile. These results provide a new reference for the future use of TTE and TEE.

First, we observed that in the evaluation of the aortic valve annulus and AVA, there were no significant differences in various detection indicators between echocardiography and MSCT, confirming the high accuracy and reference value of echocardiography as a non-invasive and convenient detection scheme. After surgery, the LVESD, IVST, PWT, PASP, AVPG_max_, AVPG_mean_, and V_max_ patients were all lower than before the operation. At the same time, LVEF and AVA were higher, indicating a significant improvement in their cardiac function. These results align with those of Peteiro J et al. [12] when investigating the changes in echocardiographic parameters before and after surgery in patients with severe AS. TAVR is known to replace the function of the aortic valve by inserting an artificial aortic valve through a catheter into the diseased aortic valve [13]. Therefore, when TAVR is used to correct severe AS, the patient’s AVPG_mean_ will be significantly reduced, leading to a decrease in resistance load, an enhancement of cardiac blood supply function, and an increase in LVEF, which is also in line with the imaging manifestations of left ventricular reverse remodelling [14]. Meanwhile, in the follow-up study of 176 patients with aortic regurgitation by Zeng Q et al. [15], it was found that the left ventricle of patients underwent evident reverse remodelling, with a smaller left ventricular inner diameter, a thinner left ventricular wall thickness, and an increased LVEF, supporting our findings.

According to research reports, more than 0.6%-4.7% of post-TAVR patients will develop moderate or severe PVL, a condition associated with an increased in-hospital mortality rate [16]. In this study, none of the patients had moderate or severe PVL due to poor valve fitting after surgery. The reasons are as follows: (1) Preoperative MSCT and echocardiography accurately evaluated the anatomical structure of the aortic root (such as aortic root diameter [AORD] and aortic annulus diameter), providing an accurate basis for clinical practice. Moreover, the artificial aortic valve model most suitable for the patient’s aortic root structure is selected for the surgery. (2) The unique skirt design of the self-expanding artificial aortic valve adopted in our hospital effectively reduces PVL. Besides, 7 cases (15%) underwent permanent pacemaker implantation due to atrioventricular block in this study. The results of the study by Elmaraezy A et al. [17] showed that TAVR was associated with a higher risk of permanent pacemaker implantation (risk ratio (RR) 2.57, 95% confidence interval (CI) [1.36, 4.86]), vascular-access complications at 1 year (RR 1.99, 95%CI [1.04, 3.80]), and paravalvular aortic regurgitation at 30 days (RR 3.90, 95% CI [1.25, 12.12]), compared to SAVR.

Regarding echocardiography, transesophageal and transthoracic approaches are currently employed in clinical practice. However, there is still a lack of reference regarding the differences in their application in heart valve replacement for severe AS. In this regard, we compared the clinical effects of the two schemes. First of all, regarding postoperative hemodynamics, SVRI, GEDVI, and ITBVI were higher in the TEE group than in the TTE group. At the same time, EVLWI was lower, suggesting that TEE is more beneficial for improving postoperative hemodynamics in patients. As for cardiac function, we observed no difference in BNP, cTnI and CK-MB between the two groups (P>0.05), indicating no significant difference in the effect of the two examination methods on the patient’s cardiac function. However, when comparing stress response, inflammatory response, and pain conditions, we see that the TEE group is better than the TTE group in all cases, indicating that TEE has a higher safety profile. TEE and TTE are invasive mechanical manoeuvres, and the body is bound to produce a stress response during the examination. However, TEE does not affect the surgical operation and visual field and can be performed simultaneously during surgery and anaesthesia without causing multiple stressful stimuli to the patient [18]. Furthermore, a TEE examination can perform targeted exhaust and determine the amount and location of gas embolism formation, which can also reduce the stress and inflammatory response of the patient to some extent [19]. In a study by Dahl A et al. [20], they also found that in patients with cardiovascular and infectious diseases, the use of TEE for examination did not further increase the inflammatory response of the patients, which also validates the high safety of TEE. It is also because TEE has a milder stress injury and inflammatory response that the patient experience of the examination is better, and therefore, the level of pain mediators is further reduced compared to TTE.

This study has the following limitations: (1) The sample size is small, and the follow-up time is short, so it is necessary to expand the sample size and extend the follow-up time in the future to confirm the results of this study. (2) MSCT was not performed on patients after surgery in this study. Further postoperative MSCT should be performed and combined with echocardiography to jointly evaluate the changes in artificial aortic valves, heart structure, and function before and after TAVR.

## Conclusion

Echocardiography has an excellent guiding effect on implementing TAVR in patients with severe AS and can assist clinicians in better completing it. In addition, TEE has a higher safety profile than TTE, reduces stress and inflammatory responses in patients, and is recommended as the first choice.

## Dodatak

### Data availability

Original data in this study are available from the corresponding author upon reasonable request.

### Acknowledgements

Not applicable.

### Conflict of interest statement

All the authors declare that they have no conflict of interest in this work.
